# Optical Coastal GNSS-Denied Navigation for Reduction in AUV Underwater Navigation Error

**DOI:** 10.3390/s26144601

**Published:** 2026-07-20

**Authors:** Tomasz Praczyk, Jacek Zalewski

**Affiliations:** Department of Computer Science, Polish Naval Academy, 00-635 Warsaw, Poland; t.praczyk@amw.gdynia.pl

**Keywords:** optical navigation, semantic segmentation, AUV, GNSS-denied environment

## Abstract

The paper addresses the problem of maritime navigation in coastal areas without access to satellite positioning data. The proposed approach relies on visual information from a monocular camera supported by map-derived coastal information. Although the operational concept assumes the use of publicly available cartographic data, the method’s preparation and evaluation require additional processing steps, including high-resolution aerial imagery, GIS-based extraction of coastal features, semantic segmentation, and trained convolutional neural network models. The problem discussed in the paper concerns, for example, Autonomous Underwater Vehicles that seek to reduce underwater dead-reckoning navigation error by surfacing and using information about what is visible around them, in a way similar to how a human would. To solve the above problem, a system was proposed that compares the camera’s representation of the observed coastline with the map representation of the area where the vehicle is most likely located. The system was validated using real-world data. The tests revealed that the information contained in a flat map is insufficient for accurate position estimation. Accuracy is also significantly affected by errors in the semantic segmentation used to extract land features from camera images, as well as by potential errors in the camera viewing angle. The achieved accuracies are sufficient for navigation away from land, but when operating close to land, the proposed system appears significantly insufficient. The paper specifies the system and reports the results.

## 1. Introduction

Autonomous Underwater Vehicles (AUVs) are a tool increasingly used for various purposes [1], e.g., to support deep-sea divers [2], monitor critical underwater infrastructure, research the marine environment, and detect and neutralise sea mines. However, operating such vehicles in an underwater environment [3] is not as easy as with flying, surface, or land drones. The first problem AUVs face compared to their surface counterparts is significant communication difficulties [4]. Radio communication is impossible underwater, and the only available option is acoustic communication [5]. However, in this case, the problem lies in the acoustic channel’s very low bandwidth.

Another challenge for AUVs in the underwater environment is navigation [6]. Drones operating over the surface of the water have easy access to information from satellite systems [7] such as the US GPS and the European GALILEO, enabling highly accurate and frequent positioning. Even if this access is difficult or impossible, continuous observation of the surrounding world [8], which is rich in characteristic objects that serve as reference points for navigation systems, is still possible. In the case of the underwater environment, where visibility is very low [9], sometimes limited to a few meters, as in the North Sea or the Baltic Sea, for example, there is no such comfort as in the case of operating in a clear surface environment [10].

Underwater navigation currently relies on three main approaches. The first approach, represented by systems such as LBL and USBL, uses external infrastructure for navigation [11]. For example, suppose that we have a system of three or four underwater “satellites” with known, unchanging positions that transmit an acoustic signal allowing us to determine the distance to the signal source. In that case, similarly to satellite systems, we can easily determine the receiver’s position. Unfortunately, such solutions can only be applied when operating in a location where our underwater “satellite” system can be placed, for example, around a critical infrastructure facility. Away from such a system, we must use another approach.

One option is to use underwater observation systems, such as sonar [12] (i.e., underwater acoustic radars). If the AUV operates near a seabed rich in recognisable landmarks, we can use them as navigational cues. For example, we can navigate along an underwater pipeline or use the SLAM method [13], which, thanks to continuous environmental observation, can enable safe navigation. But what if the seabed is flat and devoid of anything distinguishable from the background? Then we have to resort to dead reckoning.

Dead reckoning [14] is an onboard system independent of external sources of information. It is based on the measurement of speed, direction, and depth (In reality, the measurement most often does not involve speed, direction, or depth. Other parameters are measured, e.g., pressure, acceleration, and angular velocity) using onboard sensors. The problem in this case, however, is that the position error increases with the duration of the underwater mission, due to sensor imperfections. To reduce dead-reckoning navigation error, AUVs surface to utilise satellite systems. Unfortunately, in a GNSS-denied environment, satellite system support is unavailable. The only accessible information is what is visible on the surface. Far from land, this is the sun and stars. Closer to land, we can use landmarks such as urban areas, forests, beaches, isolated buildings, and harbour infrastructure.

In the simplest case, if we have image data from the area where the AUV will be operating, for example, photos of characteristic objects in the area (usually buildings) along with their precise locations, the vehicle can search for these objects and use them as reference points [15]. If at least two such objects can be found, then two bearings to these objects, determined using the onboard compass and the object-oriented camera, will be sufficient to determine the AUV’s position.

If only one such object can be detected, then, in addition to the bearing, the distance to the object is also required. For this purpose, we can use the solution proposed in [16]. It involves estimating distance by comparing the size of a standard object with the number of pixels it occupies in the image.

However, if we do not have imagery from the area where the AUV will operate, we can use the algorithm proposed in [17]. It is based on extracting the land outline from images captured by a camera around the AUV’s current position and comparing the size of the visible land, expressed in pixels, which, under the algorithm’s assumptions, corresponds to the distance to the land, with data from a digital terrain model.

It seems that one of the most challenging localisation scenarios for an AUV operating in a GNSS-denied coastal environment occurs when neither prior image data nor a terrain elevation model is available. Under such conditions, the vehicle observes the coastline from a considerable distance, making it impossible to extract distinctive landmarks or individual objects from camera images. Instead, only coarse characteristics of the visible shoreline can be observed, such as the general coastline outline and the presence of broad terrain classes, including urban areas, forests, or farmland.

This paper investigates the feasibility of using such limited visual information to estimate position in coastal environments. Rather than proposing a complete navigation solution, the study aims to evaluate which types of information contained in omnidirectional coastline observations can support localisation and which are insufficient for reliable position estimation. To this end, an Optical Coastal Navigation System (OCNS) was developed as an experimental framework for comparing omnidirectional image representations of the observed coastline with flat-map representations generated from publicly available geographic data sources, such as Google Maps.

The contribution of the paper is as follows:

1. An experimental Optical Coastal Navigation System (OCNS) for coastline-based localisation in GNSS-denied coastal regions has been developed and specified.
2. Three localisation strategies based on different combinations of angular and distance-related coastline information have been formulated and evaluated.
3. The limitations and practical applicability of flat-map-based coastline matching have been systematically investigated using real-world data.
4. The OCNS was validated using data collected in the Gulf of Gdańsk region.

The rest of the paper is as follows: Section 2 specifies the OCNS, Section 3 reports the experiments, and the final Section 4 summarises the paper.

## 2. Optical Coastal Navigation System

The OCNS consists of three modules. The first module, the Camera Representation Module (CRM), prepares a representation of the AUV’s surroundings, SR, as seen by the camera. The second module, the Map Representation Module (MRM), generates the set $S^{MR}$ of map representations MR. The OCNS assumes that the AUV knows its approximate position and the maximum position error $E_{max}^{P^{AUV}}$. The MRM generates map representations MR around the AUV’s position, estimated via dead-reckoning underwater navigation, and produces SR. All MRs are generated at positions whose distance from the estimated position does not exceed $E_{max}^{P^{AUV}}$. The final module, the Estimation Module (EM), estimates the AUV’s position by comparing the SR with all MRs generated around it. The MR, which is the most similar to SR, indicates the position of the AUV. All OCNS modules are detailed in the following sections.

### 2.1. Camera Representation Module

The purpose of this subsystem is to generate a structured representation of the environment surrounding the vehicle, denoted as SR, at the AUV’s position. The camera orientation enables the assignment of an absolute azimuth angle to individual pixel columns. This mapping allows the projection of image-based observations into a global angular reference frame. The processed image data are used to construct a compact representation of the surrounding scene, defined as follows:

(1) $$SR=\{O^{SR},H\}$$

where $O^{SR}$ denotes a set of detected areal objects grouped by semantic classes, and *H* represents the angular profile of the observed scene height.

The object component $O^{SR}$ is defined as follows:

(2) $$O^{SR}=\left\{O_{i}^{SR}\right\},\;i\in \left\{\mathrm{building},\mathrm{forest},\mathrm{other}\right\}$$

where each subset $O_{i}^{SR}$ contains areal objects belonging to a specific semantic category obtained from the segmentation process.

Each object is represented as a continuous angular segment

(3) $$o_{ij}^{SR}={\left(s_{i,j},e_{i,j}\right)}^{SR},\;s_{i,j},e_{i,j}\in \langle 0,\;360),\;e_{i,j}>s_{i,j}$$

where $s_{i,j}$ and $e_{i,j}$ denote the starting and ending azimuth angles of the *j*-th object of class *i*. This formulation assumes that each object occupies a contiguous region in the angular domain.

The set of objects of a given class is defined as follows:

(4) $$O_{i}^{SR}=\left\{o_{ij}^{SR}:o_{ij}^{SR}\cap o_{ik}^{SR}=\emptyset ,\;k\neq j,\;k,j=1,\ldots ,N_{i}^{SR}\right\}$$

which ensures that angular segments assigned to objects of the same class do not overlap. In addition to the object-based representation, a discrete height profile of the surroundings is constructed as follows:

(5) $$H=<h_{1},h_{2},\ldots ,h_{360}>$$

where $h_{\theta }$ denotes the estimated height of the observed scene (terrain or above-ground objects) in the direction corresponding to azimuth angle θ∈{1,…,360}.

The height values $h_{\theta }$ are derived from the segmentation results obtained from all processed images. For each angular direction, the corresponding pixel column is selected based on the closest azimuth match.

The resulting representation SR combines two descriptions of the environment:

- $O^{SR}$ represents detected objects as angular intervals grouped by semantic class;
- *H* provides a dense angular profile of scene height.

### 2.2. Map Representation Module

The purpose of the Map Representation Module (MRM) is to generate a structured description of the environment surrounding a given observation point, based on geospatial data sources such as a digital surface model. The representation is defined in a global angular reference frame and captures both the semantic structure of the scene and the spatial distribution of distances.

The MRM generates a set of map representations:

(6) $$S^{MR}=\left\{MR_{k}\right\}$$

where each element $MR_{k}$ corresponds to a representation constructed for a specific observation point *k* with given spatial coordinates. The structure of a single map representation MR is defined as follows:

(7) $$MR=\{O^{MR},D\}$$

where $O^{MR}$ denotes the object-based description of the surroundings, and *D* represents the angular distance profile.

The object component is expressed as follows:

(8) $$O^{MR}=\left\{O_{i}^{MR}\right\},\;i\in \left\{\mathrm{building},\mathrm{forest},\mathrm{other}\right\}$$

where each subset $O_{i}^{MR}$ contains objects belonging to a specific semantic class. The classification is derived from map data, where terrain and above-ground structures are identified and assigned to predefined categories.

Each object is represented as an angular interval:

(9) $$o_{ij}^{MR}={\left(s_{i,j},e_{i,j}\right)}^{MR},s_{i,j},e_{i,j}\in \langle 0,360),\;e_{i,j}>s_{i,j}$$

where $s_{i,j}$ and $e_{i,j}$ denote the starting and ending azimuth angles of the object, respectively. The angles satisfy constraints ensuring that each object occupies a continuous region in the angular domain. If an object spans across the $0^{\circ }/360^{\circ }$ boundary, it is represented as two separate angular intervals.

The sets of objects of a given class are defined as follows:

(10) $$O_{i}^{MR}=\left\{o_{ij}^{MR}:o_{ij}^{MR}\cap o_{ik}^{MR}=\emptyset ,\;k\neq j,\;k,j=1,\ldots ,N_{i}^{MR}\right\}$$

which guarantees that angular intervals corresponding to objects of the same class do not overlap. As a result, each direction is associated with at most one object of a given semantic category.

In addition to the object-based description, a distance profile is defined as follows:

(11) $$D=\langle d_{1},d_{2},\ldots ,d_{360}\rangle$$

where $d_{\theta }$ denotes the distance to the nearest visible object in the direction corresponding to azimuth angle θ. The profile is defined with a resolution of one degree, providing a dense representation of spatial relationships in the environment. If no object is detected along a given direction within the map extent, the corresponding value is set to $d_{\theta }=0$.

The resulting representation MR combines two descriptors:

- $O^{MR}$, representing objects as non-overlapping angular intervals grouped by semantic class;
- *D*, providing a dense angular profile of distances.

### 2.3. Estimation Module

The EM estimates the AUV position by comparing SR with MRs distributed on the map around the rough vehicle position, seeking the best match between the representations. It has three variants: EM1, EM2, and EM3. The difference between the variants lies in the comparison they make. EM1 compares $O^{SR}$ with $O^{MR}$, EM2 compares *H* with *D*, and EM3 is a combination of EM1 and EM2.

#### 2.3.1. EM1

To calculate the matching $M^{EM1}$ between $O^{SR}$ and $O^{MR}$, EM1 determines the common part of both representations $O^{C}=\left\{O_{i}^{C}\right\}$ and the differences between them $O^{D1}=\left\{O_{i}^{D1}\right\}$, $O^{D2}=\left\{O_{i}^{D2}\right\}$, where i∈{building,forest,other}. The subsets of $O_{i}^{C},\;O_{i}^{D1},\;O_{i}^{D2}$ are defined as follows:

(12) $$O_{i}^{C}\;=\;\underset{\begin{array}{c} 1\leq j\leq N_{i}^{SR}\\ 1\leq k\leq N_{i}^{MR}\\ o_{ij}^{SR}\cap o_{ik}^{MR}\neq ⌀ \end{array}}{⋃}\left(o_{ij}^{SR}\cap o_{ik}^{MR}\right).$$

(13) $$O_{i}^{D1}=O_{i}^{SR}\setminus O_{i}^{C}$$

(14) $$O_{i}^{D2}=O_{i}^{MR}\setminus O_{i}^{C}$$

The final matching is defined as follows:

(15) $$M^{EM1}\left(O^{SR},O^{MR}\right)=\frac{\sum_{i}L\left(O_{i}^{C}\right)}{1+\sum_{i}\left(L\left(O_{i}^{D1}\right)+L\left(O_{i}^{D2}\right)\right)}$$

where L(O) is the total length of all objects in *O*: $L\left(O\right)=\sum_{j}\left(e_{j}-s_{j}\right)$.

To account for possible observation angle errors during image data acquisition, as well as the range of view from a given observation point, the matching $M^{EM1}\left(O^{SR},O^{MR}\right)$ is calculated multiple times for different $O^{SR}$s and $O^{MR}$s. The final result is the best match between all tested $O^{SR}$s and $O^{MR}$s.

In the case of $O^{SR}$, various variants of this representation are tested, each rotated relative to the original by an angle in the range $<-E_{max}^{A},E_{max}^{A}>$, where $E_{max}^{A}$ is the maximum assumed observation angle error. The $O^{MR}$ modification filters out data whose distance from the map observation point at which MR is generated exceeds the assumed threshold $R^{O}$. The EM1 operation is specified by Algorithm 1.
**Algorithm 1** EM1.**Require:** $O^{SR}$, $O^{MR}$, $E_{max}^{A}$, $\Delta E^{A}$, $R_{max}^{O}$, $R_{min}^{O}$, $\Delta R^{O}$, *D***Ensure:** $M^{EM1}\left(O^{SR},O^{MR}\right)$  1:$R^{O}\leftarrow R_{min}^{O}$  2:$M_{best}\leftarrow 0$  3:**while** $R^{O}\leq R_{max}^{O}$ **do**  4:      $O_{R}^{MR}\leftarrow filter\left(O^{MR},R^{O},D\right)$▹ Remove $\left(s^{MR},e^{MR}\right)$ whose average distance to the coast exceeds $R^{O}$  5:      $shift\leftarrow -E_{max}^{A}$  6:      **while** $shift\leq E_{max}^{A}$ **do**  7:            $O_{shift}^{SR}\leftarrow rotate\left(O^{SR},shift\right)$  8:            Generate $M^{EM1}\left(O_{shift}^{SR},O_{R}^{MR}\right)$ according to Equation (15)  9:            **if** $M_{best}<M^{EM1}\left(O_{shift}^{SR},O_{R}^{MR}\right)$ **then**10:                  $M_{best}\leftarrow M^{EM1}\left(O_{shift}^{SR},O_{R}^{MR}\right)$11:            **end if**12:            $shift\leftarrow shift+\Delta E^{A}$13:      **end while**14:      $R^{O}\leftarrow R^{O}+\Delta R^{O}$15:**end while**16:**return** $M_{best}$

#### 2.3.2. EM2

EM2 uses the vector *H*, containing the pixel heights of the coastline extracted from the semantically segmented image, to derive an approximate distance estimate $\hat{D}\left(H\right)$ to the visible land. This estimate is not based on a direct geometric relationship between pixel height and range, since the true terrain elevation is generally unknown and the observed coastline profile depends on multiple factors, including the shape and local topography. Instead, the entire vector *H* is treated as an image-derived feature describing the appearance of the visible coastline from the current observation point. Although the resulting distance estimate may be inaccurate, it still provides useful information about the coastline’s relative proximity. It can therefore support navigation in GNSS-denied environments, where dead-reckoning errors may accumulate over time.

The estimate $\hat{D}\left(H\right)$ is subsequently compared with vectors *D*, each of which defines the distance to the coastline from a given map point. To estimate *D*, three regression methods are used, i.e., (i) Linear Ordinary Least Squares (LOLS), (ii) Linear Total Least Squares (LTLS) [18], and (iii) Kernel Regression (KR). The first two methods are well-known, while the KR is defined in Algorithm 2.

To build each of the regressors, training pairs $\left\{\left(h_{i}^{L},d_{i}^{L}\right)\right\}$ are used, defining the pixel height of the land $h_{i}^{L}$ and the corresponding distance to the land $d_{i}^{L}$ for viewing angle *i* degrees. These pairs are generated from sample images of the coast recorded at points with known coordinates, which allows the distance $d^{L}$ to be determined.

Obtaining an effective regressor, however, requires filtering the entire available training dataset and removing pairs that could degrade the accuracy of the estimate. The pairs that should be removed primarily correspond to semantic segmentation errors, which can generate land where it is very distant and consequently invisible, or can “lose” land where it should be visible. Another example of faulty pairs involves cases where land is erroneously elevated or lowered.
**Algorithm 2** KR.**Require:** ${\left\{\left(h_{i}^{L},d_{i}^{L}\right)\right\}}_{i=1}^{N^{L}}$ – learning set, σ, *h***Ensure:** $\hat{D}\left(h\right)$  1:S(h)←0  2:**for** i=1 to $N^{L}$ **do**  3:      $w_{i}\left(h\right)\leftarrow K\left(h-h_{i}^{L}\right)=\exp\;\left(-\frac{{\left(h-h_{i}^{L}\right)}^{2}}{2\sigma^{2}}\right)$  4:      $S\left(h\right)\leftarrow S\left(h\right)+w_{i}\left(h\right)$  5:**end for**  6:**if** S(h)>0 **then**  7:      $\hat{D}\left(h\right)=\frac{\sum_{i=1}^{N^{L}}w_{i}\left(h\right)\;d_{i}^{L}}{\sum_{i=1}^{N^{L}}w_{i}\left(h\right)}$  8:**else**  9:      $i^{*}=\arg\min_{i}\left|h-h_{i}^{L}\right|$10:      $\hat{D}\left(h\right)=d_{i^{*}}^{L}$11:**end if**12:**return** $\hat{D}\left(h\right)$

To filter the training data, EM2 employs the DBSCAN clustering algorithm [19]. It clusters the data and identifies outliers, which are then removed from the training dataset.

After filtering the training data and building a regressor, EM2 compares the vector *H* included in SR with the vectors *D*, each representing a specific position on the map and corresponding MR representation. Matching *H* to a single *D* is performed according to Algorithm 3. It compares *H* and *D* at each position *i* (viewing angle) in both vectors and returns the sum of all comparisons. There are three cases to consider when comparing vectors. The first case is when $h_{i}>0$ and $d_{i}>0$, i.e., when land is visible in the image, and the distance to the coastline is sufficiently small for the land to be visible from the observation point. In this case, the result is the absolute difference between the exact distance $d_{i}$ and the estimated distance $\hat{D}\left(h_{i}\right)$. The second case occurs when $d_{i}=0$ and $h_{i}>0$, i.e., when it is impossible to see the land because of the distance to it ($d_{i}=0$—see description of MRM, Section 2.2); however, it is visible in the image ($h_{i}>0$). The algorithm strongly punishes this case. The last case considered occurs when $d_{i}$ and $h_{i}$ agree on land invisibility, which improves the comparison result. The case in which the map indicates that the land may be visible ($d_{i}>0$), whereas the SR does not confirm this ($h_{i}=0$), is not considered by the algorithm. The reason is that $d_{i}>0$ only indicates the potential for land visibility; it does not guarantee that it is visible. In consequence, $h_{i}$ and $d_{i}$ disagree; however, in reality, they can agree.

#### 2.3.3. EM3

The EM3 is executed in two phases. First, EM2 is run, producing matchings between *H* and the set of *D*s generated at different map points scattered around the approximated AUV position. Then, the best *K* matchings are compared, and if they are very similar, making the correct decision very difficult, so EM1 is run for the *K* best MRs. The final result is the EM1 best matching.
**Algorithm 3** EM2.**Require:** *H*, *D*, regressor, $E_{max}^{EM2}$**Ensure:** $M^{EM2}\left(H,D\right)$  1:E1←0  2:E2←0  3:**for** i=1 to 360 **do**  4:      **if** $h_{i}>0$ and $d_{i}>0$ **then**  5:           $E1\leftarrow E1+|\hat{D}\left(h_{i}\right)-d_{i}|$  6:      **else if** $d_{i}=0$ and $h_{i}>0$ **then**           ▷ error in the image  7:           $E1\leftarrow E1+E_{max}^{EM2}$  8:      **else if** $d_{i}=0$ and $h_{i}=0$ **then**  9:           E2←E2+110:      **end if**11:**end for**12:**return** E2/(1+E1)

In Algorithm 4, $In_{EM2}$ denotes all additional input parameters required by EM2, such as the regressor and the maximum error value, whereas $In_{EM1}$ denotes all additional input parameters required by EM1, such as angular-error limits, rotation step, and filtering parameters. The sets D and $O^{MR}$ contain map representations generated for *N* candidate map points. Thus, each candidate point is represented by a pair $\left(D_{j},O_{j}^{MR}\right)$. EM2 is first used to rank all candidate points based on distance-related coastline information. If the best EM2 score is sufficiently better than the *K*-th score, the best EM2 candidate is accepted as the final result. Otherwise, the *K* best candidates are considered ambiguous and are re-evaluated using EM1, which exploits angular information about areal objects.
**Algorithm 4** EM3.**Require:** *H*, $D={\left\{D_{j}\right\}}_{j=1\ldots N}$, $O^{SR}$, $O^{MR}={\left\{O_{j}^{MR}\right\}}_{j=1\ldots N}$, *K*, $T^{EM3}$, $In_{EM2}$, $In_{EM1}$**Ensure:** $j_{best}$  1:$M^{EM2}\leftarrow \emptyset$  2:**for** j=1 to *N* **do**  3:      $m_{j}^{EM2}\leftarrow EM2\left(H,D_{j},In_{EM2}\right)$  4:      Add $\left(j,m_{j}^{EM2}\right)$ to $M^{EM2}$  5:**end for**  6:Sort $M^{EM2}$ in descending order according to $m^{EM2}$  7:K← indices of the *K* best elements of $M^{EM2}$  8:$(j_{1},m_{1}^{EM2})\leftarrow$ the best element of $M^{EM2}$  9:$m_{K}^{EM2}\leftarrow$ the EM2 score of the *K*-th element of $M^{EM2}$10:**if** $m_{1}^{EM2}-m_{K}^{EM2}>T^{EM3}$ **then**11:      **return** $j_{1}$12:**end if**13:$M_{best}\leftarrow 0$14:$j_{best}\leftarrow j_{1}$15:**for** each j∈K **do**16:      $m_{j}^{EM1}\leftarrow EM1\left(O^{SR},O_{j}^{MR},D_{j},In_{EM1}\right)$17:      **if** $m_{j}^{EM1}>M_{best}$ **then**18:            $M_{best}\leftarrow m_{j}^{EM1}$19:            $j_{best}\leftarrow j$20:      **end if**21:**end for**22:**return** $j_{best}$

## 3. Experiments

To evaluate the OCNS, its performance was verified using real-world data from the Gulf of Gdańsk region in Poland. The objective of the evaluation was not to determine a single average localisation error but to assess the spatial resolution at which coastline observations remain sufficiently distinctive to support reliable position estimation. Preliminary experiments using randomly distributed candidate positions yielded highly variable errors that depended on the local coastline geometry, limiting the usefulness of aggregate statistical measures. Therefore, candidate map representations were generated at predefined distances from the reference position. This methodology enables evaluation of the localisation capability of the proposed approach as a function of spatial separation. It provides practical information regarding the scale at which optical observations can be used to constrain position uncertainty in GNSS-denied environments. From an operational perspective, the obtained results can be used to determine the minimum spacing between candidate positions generated around a dead-reckoning estimate. If the spacing is significantly smaller than the method’s effective spatial resolution, the resulting map representations become too similar to be reliably distinguished. Conversely, using a spacing comparable to or larger than the estimated resolution increases the likelihood that the map representation corresponding to the actual vehicle position will be correctly identified as the best match to the observed coastline.

The verification tests compared all three position estimation methods: EM1, EM2, and EM3. The results of all tests are presented at the end of this section.

### 3.1. SR

#### 3.1.1. Dataset

**Figure 1:**
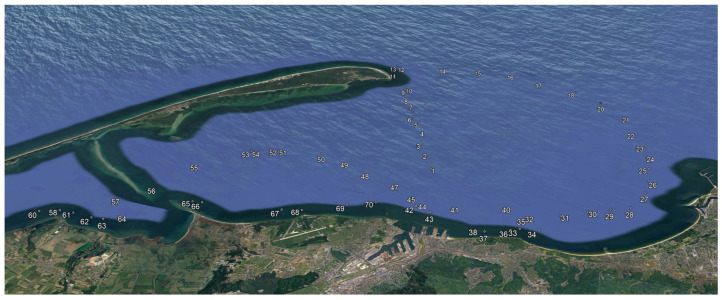
Measurement locations.

To validate the proposed method under real-world conditions, an experimental dataset was acquired consisting of 873 images collected at 70 measurement locations in the Gulf of Gdańsk—see Figure 1. For each recorded image, the camera’s geographic position and the azimuth of its optical axis were recorded, enabling precise spatial referencing of the observations.

A key parameter influencing the characteristics of the recorded images is the height of the imaging sensor above the water surface. To ensure consistency and to reflect the operational conditions of a surfaced unmanned platform, all images were acquired at a fixed height of 1 m above sea level.

The image acquisition process was carried out using a camera equipped with an integrated GNSS receiver and a digital compass. The focal length was set to a value corresponding to 25 mm for a full-frame sensor, resulting in a horizontal field of view equal to $\alpha_{h}=71.52^{\circ }$. Under these conditions, full $360^{\circ }$ coverage could theoretically be achieved using a minimum of 6 images. In practice, between 8 and 16 images were recorded at each measurement point to reduce the influence of distortions and ensure sufficient overlap between consecutive observations.

#### 3.1.2. Semantic Segmentation

To prepare SRs, semantic segmentation [20,21] was performed on the collected image dataset using the YOLOv8 Convolutional Neural Networks (CNN) [22,23]. The training of CNNs was based on another image dataset comprising approximately 10,000 high-resolution images acquired under diverse environmental conditions, supplemented with publicly available coastal imagery. To ensure reliable segmentation performance, all images were manually annotated.

Before CNN training, the images were resized and partitioned into overlapping sub-images to match the network input requirements while preserving local spatial details. To improve robustness and generalisation, the training images were augmented using random rotations, photometric transformations, and noise-based perturbations. In particular, the applied augmentation operations included image rotation, saturation modification, exposure/brightness variation, and additive noise. These transformations were selected to simulate typical changes in camera orientation, illumination conditions, atmospheric visibility, and image sensor noise that may occur during maritime image acquisition. The dataset was divided into training and validation subsets in an 8:2 ratio.

Two CNN models were designed: a multi-class model (MC-CNN) and a simplified model (SM-CNN). The MC-CNN included an extended set of object classes (e.g., vessels, offshore structures such as wind turbines, and other maritime objects), which reduced misclassification errors (e.g., classifying a ship as a building). However, these additional classes were not directly used in the final detection stage.

The SM-CNN was designed with three primary classes (sea, sky, land), where all terrain-related objects were aggregated into the land class. Additionally, during training, an auxiliary class representing objects located on the sea surface was included to reduce false detections and improve segmentation robustness, particularly in distant regions.

To semantically segment the collected images, the outputs of both CNNs were combined. When the MC-CNN provided reliable classification results, the corresponding semantic labels (building or forest) were used directly; objects belonging to the remaining classes were classified as other. In cases where these classes were not detected but the SM-CNN indicated the presence of land, the corresponding region was assigned to the class *other*, consistent with the definition $O^{SR}=\left\{O_{i}^{SR}\right\},\;i\in \left\{\mathrm{building},\mathrm{forest},\mathrm{other}\right\}$.

**Figure 2:**
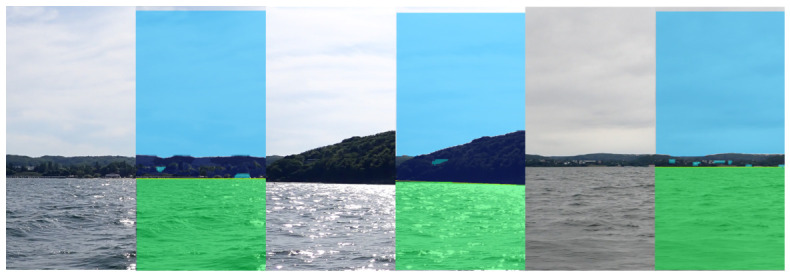
Example of image segmentation (sky—light blue, sea—green, buildings—cyan, forest-dark blue).

The quality of segmentation was evaluated in terms of its efficacy in facilitating the preparation of SRs. Illustrative results are presented in Figure 2. The SM-CNN demonstrated an effective ability to distinguish among the predominant maritime scene regions, namely the sea, sky, and land. This capability enabled the reliable localisation of the shoreline and the delineation of the sea–sky boundary. The MC-CNN provided supplementary data on the coastal zone, primarily by identifying forest and building regions that serve as reference objects in the proposed method.

The primary challenges encountered during segmentation predominantly arose in instances where the land region constituted a minuscule proportion of the scene, particularly when the shoreline was distant and manifested as a narrow strip proximate to the horizon. In such cases, the network occasionally had difficulty reliably detecting land, which could result in partial omission, fragmentation, or reduced-confidence classification. This limitation was primarily attributable to the relatively small apparent size of coastal objects in the image, rather than to errors in the segmentation of substantial sea and sky regions.

#### 3.1.3. *SR* Design

Based on segmented images, a spatial representation SR was constructed for each measurement point. The representation was defined with an angular resolution of $R=1^{\circ }$, resulting in a discretized form $SR=<h_{1},h_{2},\ldots ,h_{360}>$, consistent with the definition of the observation vector *H*.

### 3.2. MR

The generation of the MRs requires the use of geospatial data describing the area in which the system operates. In contrast to the previous approach based on elevation models [17], this study employs high-resolution aerial imagery as the primary source of spatial information.

**Figure 3:**
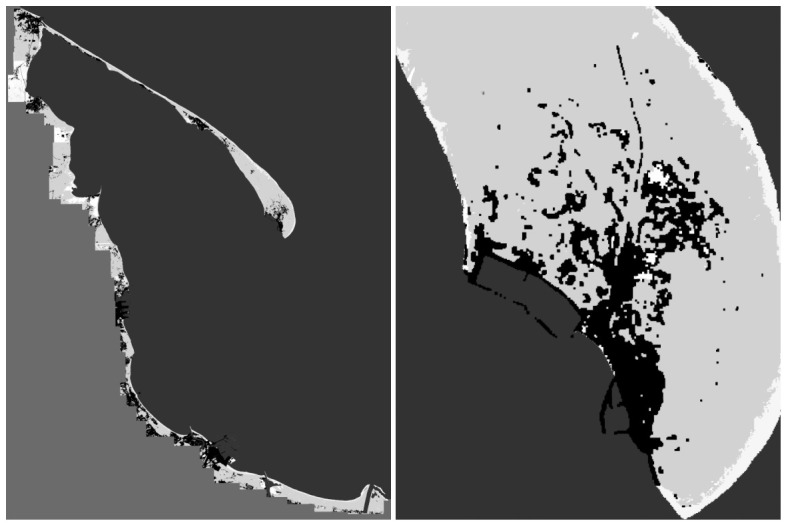
Grayscale map after segmentation in GIS software (buildings—black, forest—light grey, other land regions—white)—(**left**); an enlarged section—(**right**).

The MRs were constructed by integrating multiple high-resolution images using dedicated geographic information system (GIS) software ArcGIS Pro 3.6 into a single, spatially consistent mosaic. The source GIS data were georeferenced and processed in the WGS84 geodetic reference system, ensuring a common spatial reference for all input layers and reference objects. For map visualisation and generation of the mosaic presented in Figure 3, the data were projected into the WGS84/UTM zone 34N coordinate system. This projection was selected because it is appropriate for the Gulf of Gdańsk region and, as a conformal projection, preserves local angular relationships while limiting distortions over the analysed area. The resulting image had a resolution of approximately (40,000×30,000) pixels, covering an area of about (2500 km^2^), corresponding to the region of interest considered in the experimental evaluation.

To ensure consistency with the SR, the assembled dataset was subjected to a segmentation process within the GIS environment. The segmentation distinguished three classes: buildings (urbanised areas), forest, and other land regions. This classification scheme directly reflects the structure of the set $O^{MR}=\left\{O_{i}^{MR}\right\}$, where i∈{building,forest,other}, thus maintaining semantic compatibility between the map-based and image-based representations.

Due to the large size of the generated dataset, an additional preprocessing step was applied to reduce storage requirements and improve computational efficiency. The segmented image was converted to grayscale, preserving class information while significantly reducing data volume. As a result (Figure 3), the final high-resolution 8-bit image occupied approximately 0.9 GB of storage, which was sufficiently compact to enable efficient processing and the generation of map representations MR for subsequent analysis.

The SRs were constructed at a set of predefined observation points, which served as reference locations for the analysis. At each reference location, a corresponding map representation, denoted as $MR_{i=0}$, was also generated. Furthermore, five additional MR sets were created: MR-1.6, MR-2.4, MR-3.2, MR-4, and MR-8. Each set differed in the distance $D^{MR}$ = 1.6, 2.4, 3.2, 4, and 8 km between the reference position and the positions of the generated MRs. All MRs belonging to a given set were located at the same distance $D^{MR}$ from the reference position.

The purpose of introducing multiple $D^{MR}$ values was to analyse how the spatial separation between candidate positions affects the distinguishability of their corresponding map representations. When candidate positions are located close to one another, their associated (MR)s may be highly similar, making reliable identification of the correct position difficult even when the matching algorithm performs correctly. By gradually increasing $D^{MR}$, the generated map representations become more distinct, allowing the practical localisation capability of the method to be evaluated as a function of the spatial separation between candidate positions rather than through a single aggregate error metric.

Each set of map representations contained a maximum of 37 MRs, i.e., one MR generated at the reference position ($MR\text{-}D_{i=0}^{MR}$) and up to 36 additional MRs ($MR\text{-}D_{i=1\ldots 36}^{MR}$) evenly distributed every 10 degrees on a circle of radius $D^{MR}$. Candidate positions located on land were omitted.

**Figure 4:**
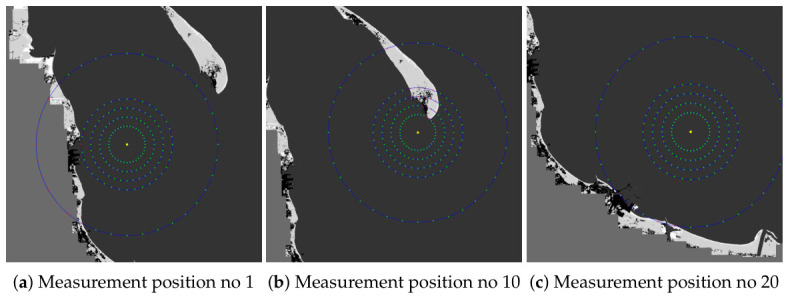
Distribution of candidate points for MR-1.6, MR-2.4, MR-3.2, MR-4, and MR-8 for three example measurement positions.

Example spatial distributions of $MR\text{-}D_{i=0\ldots 36}^{MR}$ are illustrated in Figure 4, where points located on land (and thus discarded) are marked in red, while correctly identified sea points are shown in green.

For each candidate point, the map representation MR was generated by analysing the map in all azimuth directions with a resolution of $1^{\circ }$, resulting in a distance profile $D=\langle d_{1},d_{2},\ldots ,d_{360}\rangle$. If land was not visible in a given direction, then $d_{i}=0$; otherwise $d_{i}>0$. Distances were calculated using the map resolution of approximately 1 m per pixel.

### 3.3. Results

The accuracy of the EMs was estimated separately for each $MR\text{-}D^{MR}$. The accuracy for one $MR\text{-}D^{MR}$ was calculated as the number of SRs out of all 70 SRs that were correctly associated with the correct MR, i.e., with $MR_{i=0}$ generated in the SR position. Formally, the EM evaluation can be written as follows:

(16) $$F\left(EM,MR\text{-}D^{MR},SR\right)=\sum_{i=1}^{70}\frac{\phi (SR_{i},MR\text{-}D_{i}^{MR})}{70}$$

(17) $$\phi \left(SR,MR\text{-}D^{MR}\right)=\left\{\begin{array}{cc} 1 & \mathrm{if}\;\arg\max_{j=0,\ldots ,36}M^{EM}\left(SR,MR\text{-}D_{j}^{MR}\right)=0\\ 0 & \mathrm{otherwise} \end{array}\right.$$

#### 3.3.1. EM1

The parameter setting for EM1 was as follows: $E_{max}^{A}$ = 5, and 10 deg, $\Delta E^{A}$ = 1 deg, $R_{max}^{O}$ = 8, 16 and 24 km, $R_{min}^{O}$ = 6 km, $\Delta R^{O}$ = 2 km, $O^{MR}=\left\{O_{building}^{MR}\right\}$, $\left\{O_{building}^{MR},O_{forest}^{MR}\right\}$, and $\left\{O_{building}^{MR},O_{forest}^{MR},O_{other}^{MR}\right\}$.

EM1 testing started with MR-8 and ended with MR-4 due to highly unsatisfactory results. In the case of MR-8, the best result was F(EM1,MR-8) = 0.45, i.e., only 32 correct decisions, identifying the SR position as the measurement position. This result was obtained for $O^{MR}=\left\{O_{building}^{MR}\right\}$, $E_{max}^{A}$ = 5 deg, and $R_{max}^{O}$ = 16 km. In other cases, i.e., when the map also included forests or other areas, the results were even worse. In the case of MR-4, and once again the best option, $O^{MR}=\left\{O_{building}^{MR}\right\}$, only 20 correct decisions were obtained, yielding the result F(EM1,MR-4) = 0.28, and demonstrating the very low accuracy of EM1, which decreases with decreasing distance $D^{MR}$.

There are several reasons for this. One is, of course, the erroneous operation of semantic segmentation, which either fails to “see” certain areas or artificially creates them in the image. Analysis of the segmented images, however, indicates that the impact of segmentation errors is not decisive.

**Figure 5:**
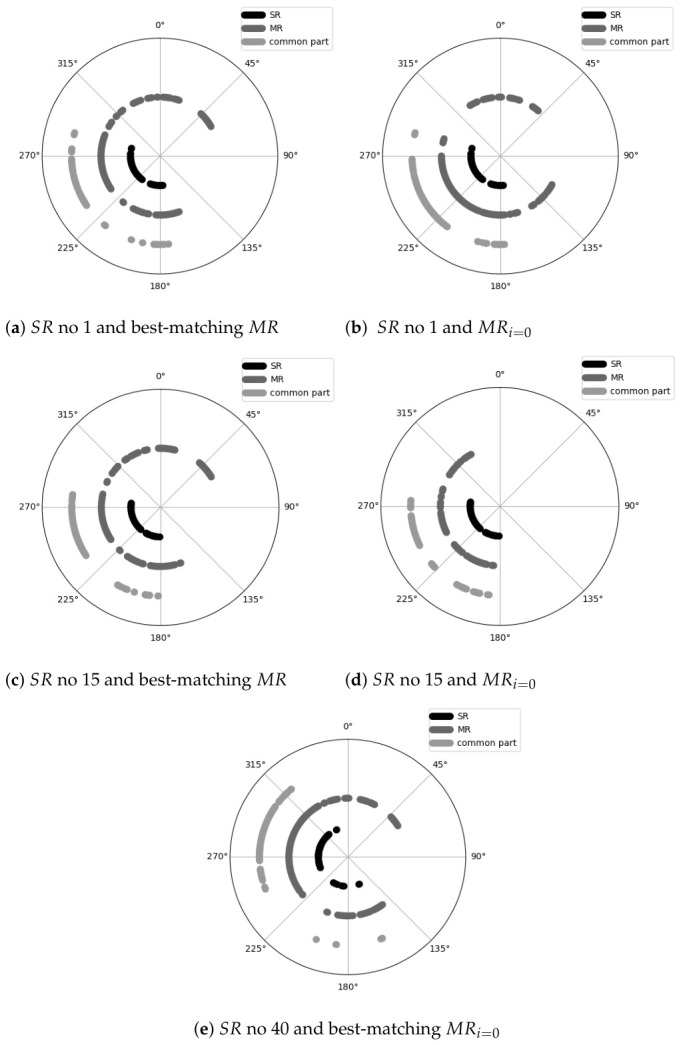
Example results of EM1 and $O^{MR}=\left\{O_{building}^{MR}\right\}$—the graphs show SR, MR, and their common part; the first four graphs illustrate errors of EM1, while the last graph shows the correct EM1 decision.

Another reason for the poor EM1 results is potential compass errors in the camera. During testing, the maximum error was first assumed to be $E_{max}^{A}$ = 5 deg, then increased to 10 deg. Increasing $R_{max}^{A}$ at first glance seems to be the best way to eliminate compass error. The problem, however, was that increasing $E_{max}^{A}$ to 10 deg resulted in even more frequent association of SR and MR objects (usually urbanised areas), even though these did not correspond. In most cases, EM1, “looking” further and further by gradually increasing $R^{O}$ (see Algorithm 1), could match SR with many good MRs even though these MRs were located far from each other. Often, the difference between the best match $M_{best}^{EM1}$ and the other matches was negligible, as shown in Figure 5.

The first two lines in this figure show a situation in which EM1 made an incorrect decision, associating SR with the incorrect winning MRs. Figure 5a,c show the matchings of SR with winning MRs, while Figure 5b,d show SRs together with $MR_{i=0}$, i.e., MRs generated on the map at the location where SR was recorded. In addition to SRs and MRs, the figures also show the common parts between the two representations. It can be seen that the differences in the common parts between the winning MRs and $MR_{i=0}$ are very small.

In some cases, $MR_{i=0}$ was a winning MR, but subsequent analysis of matches between individual SR and $MR_{i=0}$ objects showed that they refer to objects that are actually distant from each other.

It seems that the main factor leading to difficulties in pairing SR with the correct $MR_{i=0}$ is the lack of information about the distances to objects observed in SR. The lack of this information causes Algorithm 1 to search for increasingly distant SR matches on the map and often associates SR with objects that are either inland or cannot be seen from the sea surface due to the large distance. The solution may be to limit $R_{max}^{O}$. However, the experiments revealed that this solution causes EM1 to miss many objects, resulting in even worse performance.

#### 3.3.2. EM2

Testing this variant of position estimation required training images of the coast for regressors designed to estimate distance to land from visual data. These training images were recorded in the Gulf of Gdańsk, like the other images used in the experiments. The training set consisted of 86 images of the coast recorded at various positions in the Gulf. These images were then semantically segmented to extract land and determine its pixel height. The pixel values were then associated with distances to land, which was possible given information about the recording position and the camera viewing angle. After creating the training set, it was filtered using DBSCAN (eps = 0.7, min_samples = 6), and the resulting data were used to construct the aforementioned regressors, which are core components of EM2 and Algorithm 3.

This variant of the estimation module proved to be more accurate than EM1, as shown in Table 1. The best option turned out to be Kernel Regression (KR(σ=3); see Algorithm 2). For higher $D^{MR}$, this variant achieved the following results: F(EM2,MR-4) = 0.83 and F(EM2,MR-8) = 0.86. Unfortunately, for smaller $D^{MR}$ values, EM2 still makes many mistakes. For MR-3.2 and MR-2.4, slightly less than half of the decisions are incorrect, and for MR-1.6, most decisions are incorrect.

**Figure 6:**
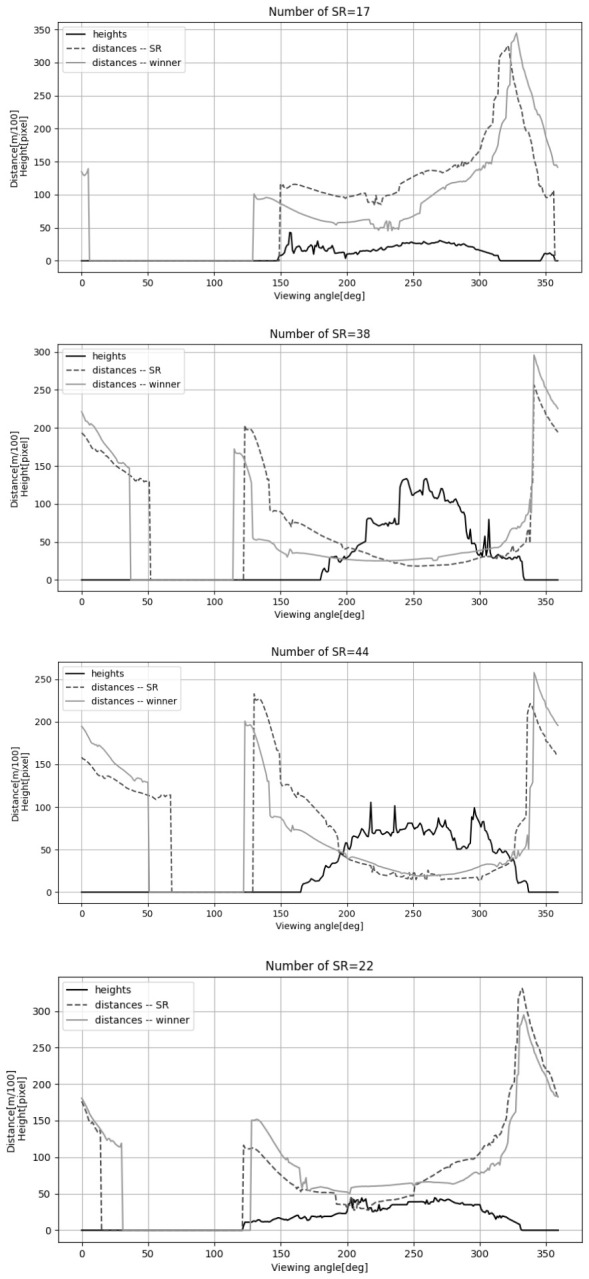
Examples of EM2 errors.

An example of EM2 malfunction for MR-4 is shown in Figure 6. This figure shows the four situations in which EM2 made the largest errors, i.e., those in which the matching between the SR and $MR_{i=0}$ differed significantly from the best matching. The graphs show the pixel height *H* representing the SR, the distance to land *D* representing the $MR_{i=0}$, and the distance *D* representing the best-matching MR.

Analysis of Figure 6 shows two potential causes of errors. In the first upper graph, where the EM2 error is highest, the most likely cause is incorrect distance estimation by the KR. For the entire angular range for which land is visible on the SR, its height is low, suggesting a large distance to land, and this is, in fact, the case. Distance *D* at the SR position for the land visibility range is approximately 10 km. Meanwhile, the winning MR, that is, the MR best matched with SR, is approximately 4–5 km closer. This means that KR found the distance to land to be closer. The reason is that the training data used to construct the KR were closer to the decision corresponding to the winning MR than $MR_{i=0}$. The situation presented in the upper graph must simply differ from what we most often observe in the Gulf of Gdańsk. A land height of approximately 30 pixels most often corresponds to a distance of about 5 km, not 10 km.

A different situation occurs in the remaining graphs, where the EM2 error is lower. In this case, it turns out that for the area in which KR is applied, the best-matching MRs, even though they are 4 km away from SR, are very similar to $MR_{i=0}$.

#### 3.3.3. EM3

**Figure 7:**
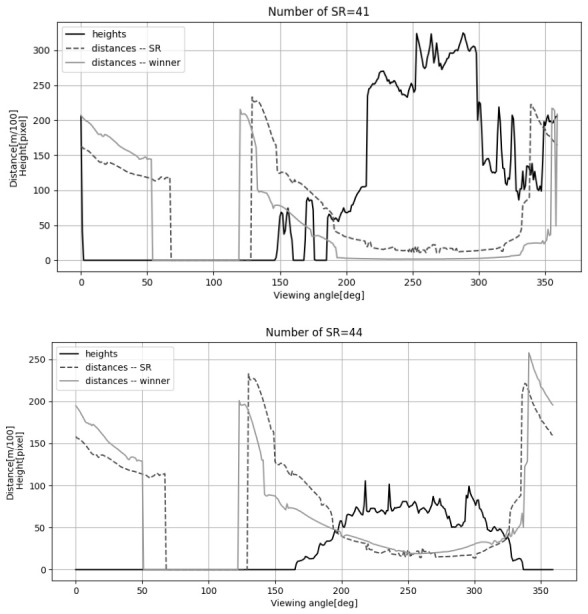
Two cases misclassified by EM2 and correctly classified by EM3.

EM3 was run for K=4, $T^{EM3}=0.00005$ and $E_{max}^{E}M2=$ 10,000 and the best EM2 variant with KR regressor. It turned out that adding EM1 to EM2 in situations where multiple MRs achieved similar matching scores slightly improved the results of the original EM2 variant, but only for MR-4 and MR-8. The results obtained for these cases are as follows: F(EM3,MR-4) = 0.86, F(EM3,MR-8) = 0.9. For MR-4, improvement occurred for 2 SRs, and for MR-8, for 3 SRs. For MR-4, both correctly classified cases were previously incorrectly classified. They are shown in Figure 7 along with the best-matching MRs chosen by EM2. For MR-8, improvement was observed for previously misclassified SRs in 4 cases, and 1 previously correctly classified SR was assigned to an incorrect MR.

## 4. Summary

This paper investigated the feasibility of using optical observations of the coastline to estimate position in GNSS-denied maritime environments. Three localisation approaches were evaluated.

The first approach, EM1, relied solely on the angular arrangement of areal objects visible on both maps and camera images. Experimental results demonstrated that this approach was ineffective because angular information alone was insufficient to uniquely associate observed objects with their map counterparts in areas containing numerous visually similar features.

The second approach, EM2, incorporated an estimate of the distance to the coastline derived from image features describing the visible land. This additional information significantly improved localisation performance compared with EM1, demonstrating that approximate range-related information plays a critical role in coastline-based localisation. However, the achieved localisation resolution remained limited. The experimental results indicate that the method can reliably discriminate between candidate positions separated by approximately 4 km, which is insufficient for precise coastal navigation.

The third approach, EM3, combined the distance-related information used by EM2 with the angular information employed by EM1. In ambiguous situations, angular observations were used as an additional decision criterion. This approach provided a modest improvement over EM2, increasing the probability of correctly identifying the reference position, but without fundamentally changing the achievable localisation resolution.

The obtained results indicate that the proposed Optical Coastal Navigation System should not be considered a replacement for conventional navigation systems in applications requiring precise coastal positioning. Instead, its current capabilities suggest potential use as a coarse localisation or position-correction mechanism in GNSS-denied environments, where even approximate position information may help constrain accumulated navigation errors and reduce the search area for more accurate localisation methods.

The experiments also demonstrated that reliable estimation of the distance to the visible coastline is the key factor limiting localisation performance. Therefore, future research will focus on improving distance estimation using additional image features. In particular, texture characteristics and the roughness of the extracted coastline silhouette will be investigated, as both features are expected to exhibit distance-dependent behaviour that may improve the discriminative capability of the proposed localisation framework.

## Figures and Tables

**Table 1:** Results of EM2—% of correct decisions, a correct decision is matching SR with $MR_{i=0}$.

	LOLS	LTLS	KR
MR-1.6	28	34	41
MR-2.4	36	40	55
MR-3.2	42	45	64
MR-4	48	48	83
MR-8	55	58	86

## Data Availability

The data that support the findings of this study are available from the corresponding author, J.Z., upon reasonable request.

## Abbreviations

The following abbreviations are used in this manuscript:

AUV	Autonomous Underwater Vehicle
LBL	Long Baseline
USBL	Ultra Short Baseline
GPS	Global Positioning System
GNSS	Global Navigation Satellite System
OCNS	Optical Coastal Navigation System
CRM	Camera Representation Module
MRM	Map Representation Module
EM	Estimation Module
MR	Map Representation
SR	Surrounding Representation
